# A protocol for developing core outcome sets for laparoscopic hiatal hernia repair

**DOI:** 10.1186/s13063-022-06845-1

**Published:** 2022-10-27

**Authors:** Xiaoli Liu, Qiuyue Ma, Jie Chen, Huiqi Yang

**Affiliations:** Department of Hernia and Abdominal Wall Surgery, Beijing Chao-Yang Hospital, Capital Medical University, Beijing, China

**Keywords:** Laparoscopic hiatal hernia repair, Core outcome sets, Interviews, Delphi study, Scoping review

## Abstract

**Background:**

Hiatal hernias negatively damage patients’ health and life quality. Laparoscopic hiatal hernia repair is currently the gold standard for the treatment of hiatal hernia (LHHR). Numerous clinical trials on laparoscopic hiatal hernia repair have been done, but the published findings are highly variable due to the lack of unique outcome sets. Basic outcome sets have ever been established over the previous decade for a few procedures, but not for hiatal hernia repair yet. This protocol outlines the procedure to develop a core outcome set for laparoscopic hiatal hernia repair COS-LHHR). COS-LHHR will provide a unique criteria for clinical investigations.

**Methods:**

This study will be conducted in four phases: (1) scoping reviews of existing qualitative studies and outcome reporting in randomized controlled trials to develop a list of potential outcome domains; (2) qualitative interviews with patients to explore the impact of laparoscopic hiatal hernia repair and the outcomes that they care most; (3) a multi-round e-Delphi study to achieve preliminary consensus on the core outcome set; and (4) an evidence-based consensus on a core outcome set will be achieved through a structured group consensus meeting, recommending best assessment outcome sets.

**Discussion:**

The development the COS-LHHR will guide clinical research of laparoscopic hiatal hernia repair with unique outcome assessment. This would improve comparative analyses among studies.

## Introduction

A hiatal hernia (HH) occurs when the stomach or abdominal viscera protrude from the abdominal cavity and into the thoracic cavity through hiatus [1]. The symptoms are usually intermittent substernal pain, early satiety, dysphagia, and anemia. Acute volvulus may cause tissue ischemia, necrosis, and systemic sepsis in serious cases [2]. To some extent, these disorders harm patients’ health and have an impact on their life quality [3, 4].

The gold standard for the treatment of hiatal hernia is laparoscopic hiatal hernia repair (LHHR) [5–7]. A vast number of clinical trials have been conducted on laparoscopic hiatal hernia repair, but the results reported are highly heterogeneous. The key outcome indicator of most clinical research is the recurrence rate, while the definition of recurrence rate varies. Some studies use radiological recurrence, but others use anatomical recurrence [3, 6, 8, 9]. The outcome comprised secondary outcomes in addition to the primary result, and reporting of additional outcomes was even more inconsistent. Some studies provide readmission rates, follow-up times, and postoperative clinical symptoms, whereas others record reintervention rates, postoperative acid secretion inhibitor use, clinical symptom scores, and surgical revision [5, 7, 9, 10]. Inconsistency in the reporting of outcome hinders study such as systematic reviews or meta-analyses. Therefore, the construction of standardized outcome indicators is of great value for standardized clinical research.

Standardized outcome indicators should include both objective and subjective indicators. Objective indicators are based on observable and quantitative factors and subjective indicators use people’s own evaluation of their satisfaction with their lives before and after surgery-a cognitive evaluation of their entire lives [11, 12]. Outcomes in existing studies have focused more on objective indicators such as recurrence and complication rates. Clinical efficacy evaluation solely comprises objective signs such as esophageal manometry, esophageal PH value monitoring, and gastroscopy [1]. However, if the patient is satisfied with the treatment outcome, postoperative food, and nursing, and so on, are all factors to consider [13]. Relevant signs, such as subjective markers of status and subjective difficulties, are severely lacking in the diagnosis and treatment process. Recent concept lay a higher emphasis on alleviating patients’ symptoms and restoring digestive function, while there is no standardized evaluation indication system yet [3, 13–15]. It is intuitively necessary to integrate patient self-reported outcome indicators on the basis of current clinical effect evaluation indicators when establishing a hospital service organization system of “patient-centered care” [16]. According to reports, there is no unified evaluation index for laparoscopic hiatal hernia repair that incorporates objective indicators and subjective patient feelings. This hinders the inclusion of research findings in systematic reviews or meta-analyses, which has a significant impact on the quality of care. Consequently, it is essential to develop subjective and objective outcome indicators for laparoscopic hiatal hernia surgery.

The core outcome sets (COSs) represents standardized outcome sets that are essential to be measured [17]. COS is an agreed standardized set of outcomes that should be measured and reported, as a minimum, in all clinical trials in specific areas of health or health care [18]. The development of COSs for evaluating laparoscopic hiatal hernia repair would minimize heterogeneity in outcome measurement. The purpose of this study is to provide a set of evidence-based, consensus-based core outcomes that can be used in all studies evaluating the efficacy of laparoscopic hiatal hernia repair.

## Methods

**Fig. 1:**
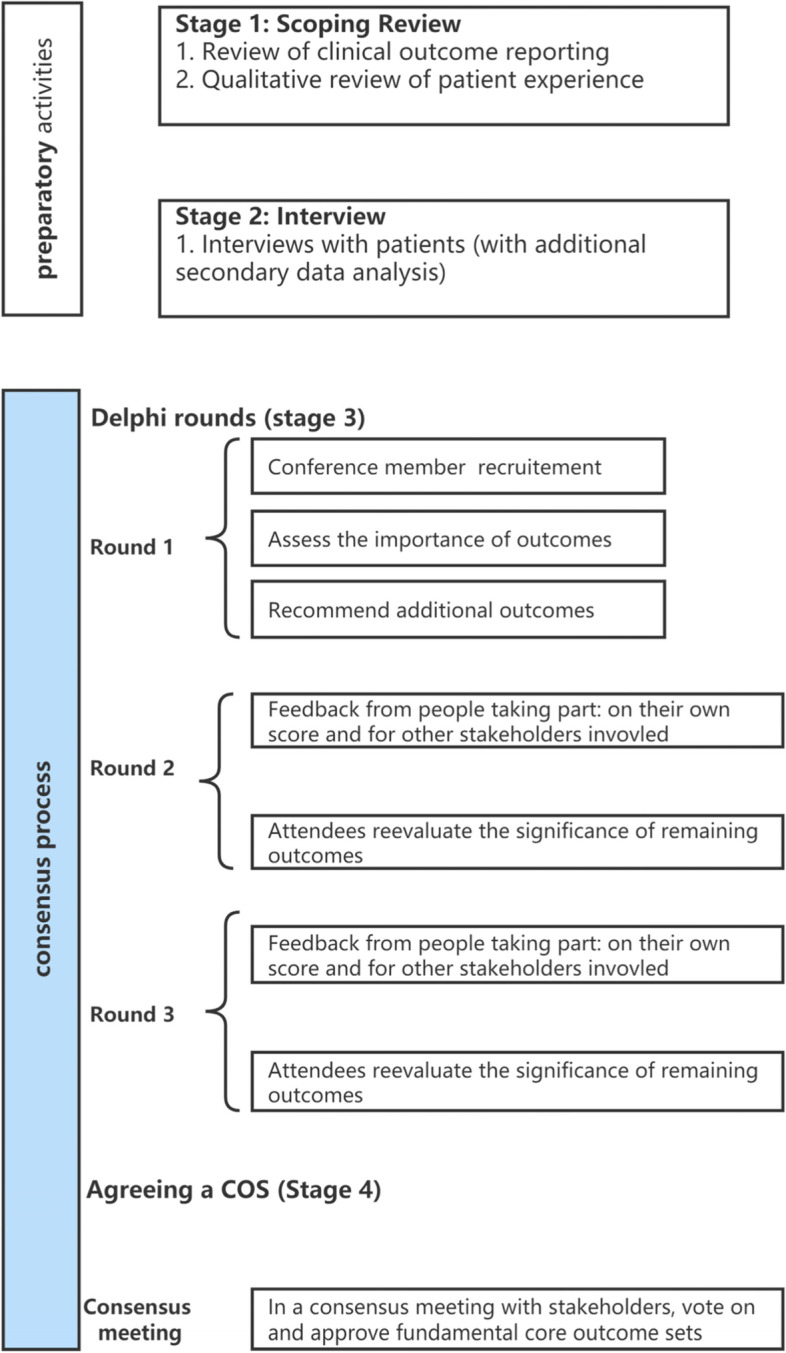
Flow diagram illustrating the process of developing COS-LHHR

The COMET initiative has registered our COS development plan (https://www.comet-initiative.org/Studies/Details/2068). The ethics committee of Beijing Chao-yang Hospital, Capital Medical University, approved the study before proceeding. A multistep method will be used to generate a consensus set of core outcome sets (COSs) for laparoscopic hiatal hernia repair in accordance with published recommendations (Fig. 1):

- A scoping review of current outcomes reported in LHHR in patients with hiatal hernia in the last 2–3 years, with the goal of establishing a list of outcome sets for LHHR.
- Qualitative interviews are mostly face-to-face personal in-depth interviews with patients to develop a set of outcome indicators that are important to patients, as well as to supplement and improve the existing list of indicators.

Three-round e-Delphi survey:

- Prioritize results through a three-round e-Delphi survey.
- Ratification of COS by a consensus conference of clinicians, methodological specialists, patients and nurses, and e-survey participants.

A consensus meeting to agree the core outcome sets of laparoscopic hiatal hernia repair in patients with hiatal hernia.

The Core Outcome Set Standards for Reporting (COS-STAR) will be used once the project is finished.

### Scope of core outcome set

This COS will be solely focused on clinical outcomes including patient-reported outcomes in patients with hiatal hernia who have undergone laparoscopic hiatal hernia repair. The scoping review is a review of quantitative studies. People over the age of 18 will be included in the population.

### Stage 1: Establishing the outcomes of LHHR

The following approaches for generating a list of LHHR outcome domains, identifying what aspects of LHHR should be measured in all scientific studies, are presented as a proportionate and expeditious approach. We anticipate duplication in studies that evaluate the same outcomes. We will not re-record duplicated results. When continued searching yields no new results, the search will end.

#### Search strategy

Electronic searches on the Cochrane Database of Systematic Reviews, PubMed, Medline, EMBASE, the World Health Organization International Clinical Trials Registry Platform for ongoing trials, CNKI, and Wanfang data will be used to identify LHHR outcome domains from systematic reviews and primary research studies. An information specialist (MQY) will conduct the electronic searches, with the findings being entered into the NoteExpress reference manager. Both controlled headings (e.g., medical subject headings (MeSH)) and free text will be used in the plan. Concepts will be used to organize terms and combine them. The search methods will be documented and reported on. The dates of searches will be logged and the search results will be managed in reference management software.

#### Types of studies

Publications that focus on hiatal hernia patients and include clinical trials, observational studies, and systematic reviews of other trials are eligible. We will only include quantitative studies, excluding qualitative and mixed-method studies. A list of outcomes will be compiled, including both LHHR specificity for hiatal hernia and patient-reported outcomes.

#### Types of interventions

We will add any paper in which laparoscopic hiatal hernia repair is a treatment.

#### Types of participants

The study population was patients with hiatal hernia.

#### Exclusion criteria

- 1) There is no full text in Chinese or English;
- 2) The impossibility to access the whole text;
- 3) Basic research, mechanism studies, and animal studies;
- 4) Research into Chinese medicine;
- 5) Abstracts from conferences, expert opinions, research proposals, book chapters, and current events.

#### Deciding eligibility

The researcher conducting the study will evaluate titles and abstracts and select references. If there are any questions regarding eligibility, the final determination will be made in cooperation with other members of the study team. We do not suggest a two-person screening since the additional resources are not warranted by the risk of missing outcome domains, and we anticipate significant redundancy. A “Preferred reporting items for systematic reviews and meta-analyses” (PRISMA) flowchart will be utilized to document each stage of the study selection procedure [19].

#### Data extraction

The researcher will note each outcome domain measured in an eligible manuscript and the instrument or method utilized to measure. We will record author details, year and journal of publication, hiatal hernia definition, participant characteristics, the type of intervention utilized, reported outcomes, and outcome definition(s). A second reviewer verifies the extracted data’s accuracy as part of quality assurance.

#### Coding, averaging, and classifying outcomes

In a spreadsheet, outcome domains will be included and categorized according to the taxonomy established by the COMET handbook [18, 20]. And the systematic review will identify all outcomes that have been reported and the frequency of reporting.

Members of the advisory panels (including clinical experts in the field of hiatal hernia-two chief physicians, two associate chief physicians, one attending physician, and one resident, as well as one researcher in clinical methodology and one researcher in epidemiology and health statistics) will convene to evaluate and discuss the first list of outcomes and may offer suggestions for adding outcomes, aggregating outcomes, or modifying terminology and descriptions for each result to make them language-accessible.

### Stage 2: Complementing the outcomes of LHHR surgical intervention in individuals with HH

Patient-reported outcomes were included in the process of establishing the COS of LHHR. Studies have shown that excluding patients from the study process can result in the omission of vital findings. Consequently, it is of the utmost necessity to incorporate patient-reported indications into the COS when the indicator sets are being developed.

We will conduct semi-structured interviews with patients undergoing LHHR. This sample size is based on the information saturation principle to determine the number of patients that will be questioned. The so-called information saturation occurs when no new information is generated during an interview, at which point the information is deemed to be saturated and the interview is terminated. The interviews will be guided by a schedule co-created by the research team and patient partners (Table 1) and are expected to last up to one hour. Every interview will be audio recorded and transcribed word-for-word. During and after the interview, notes will be collected to help investigation, understanding, and reflections.

**Table 1:** Interview outline questions and tips

**Interview outline questions and tips**
**The essential issues will be:**
1. Tell me about your health
2. What is important in terms of your health?
3. What has your life been like since you were diagnosed with a hiatal hernia? Prompts: Tell me more about that, how did you feel, and what were your thoughts?
4. How do you know when a treatment is working? How important is your recovery from the hiatal hernia to you? Prompts: What is the most important thing to you? What are your expectations?
5. Other patients have talked about ‘returning to normal’. What does that mean to you? What do you care about the outcomes after surgery? What are you most concerned about after the operation? What information or content would you like to receive from your doctor after surgery? Prompts: Was the surgical procedure successful? How is the stomach able to recover? When can I resume normal eating?
6. What makes you feel well (or better)?
**Other questions, such as the following, may be valuable for guiding the interview:**
Could you describe how you acquired the hiatal hernia?
Your stomach is disturbed, and how do you often treat this condition?
- Work
- Personal and social life
- Feelings and mood
What was your most difficult time for you?
How are you feeling/ what are you thinking/ about your recovery?
How has your illness affected you?
Are there things you used to be able to perform that you no longer can?
Did you have any issues or anxieties regarding your hiatal hernia or recovery?

The sampling criteria will include treatment technique, kind of hiatal hernia, duration of acid reflux, age, and health insurance status. The sample population will be as diverse as feasible in order to capture a wide range of perspectives in the interviews. Participants will be at least 18 years old, will have undergone laparoscopic hiatal hernia surgery, and will be fluent in Chinese (written and verbal). Participants will be identified using the local hospital’s clinical register. Due to the escalating COVID-19 scenario, interviews will be performed in person, digitally (by webcam), or over the phone, based on safety recommendations. Prior to the interview, the researchers will be unaware of the patients’ identities and the purpose and significance of the study should be explained to the interviewer, and the patient’s consent should be obtained before the interview.

The interviews will be conducted and coded by LXL, MQY, and YHQ, who are not normally involved in patient care. LXL is a female research fellow with a doctorate in Clinical Research Methodology who has utilized a variety of methodologies. MQY holds a doctorate in epidemiology and is a seasoned qualitative researcher. YHQ is a doctorate-holding female surgeon who specializes in hiatal hernia surgery. The interviews will employ content analysis and thematic analysis to construct a framework for patient outcomes. The framework was then applied to the transcripts, which were indexed using NVivo software according to themes and subthemes. LXL/MQY independently indexed (coded) transcripts to evaluate their exhaustiveness and consistency. Through group talks, differences in interpretation between the researchers were overcome. The indexed data were then summarized on a thematic table that contained topics, subtopics, and patient interview content for each subtopic. On the table were also noted the frequency and depth of conversation on a particular topic or theme. Following the preliminary analysis, the research team held a number of seminars to refine and summarize the content of the topic table, improve the outcome given by the patients, and create an index list for the patients.

### Stage 3: Achieving outcome prioritization and core domain confirmation in a multi-stakeholder e-Delphi study

Delphi studies utilize a process of sequential questionnaire completion and feedback to establish expert consensus between a panel of experts [21, 22]. To ensure that the COS-LHHR reflects the perspectives of experts in the field of hiatal hernia, two panels will be defined: (1) patients who have experienced a hiatal hernia and (2) health professionals and researchers who are active in this field, representative of their professional groups, and well-positioned to implement the COS-LHHR recommendations [23].

Consensus or accepted standards for sample sizes for Delphi studies are currently lacking with expert panel sizes described in COS development ranging from 15 to over 200 panelists [24–26]. According to the actual situation, we initially determined the number of members of the expert group to be 15 to 20 people. Eligibility criteria for panelists are presented in Table 2.

**Table 2:** Eligibility criteria for participants in the e-Delphi

	Inclusion criteria	Exclusion criteria
Generic criteria (all)	Aged 18 years or older; Willing to participate in a multi-round online Delphi study; Proficient in Chinese	Unable to access a computer or digital device for the duration of the study
Expert panel 1 (patients)	Has experienced laparoscopic hiatal hernia repair within the last 2 years (at the point of contact)	Patients with hiatal hernia who did not undergo laparoscopic surgery
Expert panel 2 (professionals)	Has experience working, or conducting research, with patients undergoing laparoscopic hiatal hernia repair	Limited experience (less than 9 months) working in hiatal hernia care and no experience of working with laparoscopic hiatal hernia repair

Expert panel 1: We will identify patients—18-year-old or older adults with hiatal hernia undergoing laparoscopic hiatal hernia repair within 2 years of the e-Delphis—using the recruitment strategy outlined in stage 2. This will be a sample from China. A list of “pre-agreed” individuals will be compiled and invited to take part in the e-Delphi.

Expert panel 2: A group of health professionals (clinicians/surgeons, nurses) and researchers (trialists, reviewers, measurement experts) who are actively involved in delivering hiatal hernia care or in hiatal hernia research of relevance to laparoscopic hiatal hernia repair will be identified through national professional networks (e.g., Tencent Conference) and societies (e.g., Hiatus Hernia Group of Chinese Medical Association); published research and clinical methods of recruitment, such as snowballing and personal contacts, will be used in addition to these methods. E-mails will be sent to those who have the potential to take part in the study asking them to think about doing so. A list of people who have already reached an agreement will be compiled and asked to take part in the e-Delphi.

#### Modified e-Delphi method

The software COMET DelphiManager will be utilized in order to run the customized version of e-Delphi (University of Liverpool). It will consist of three rounds that follow one another with the same group of panelists in each round: participants who finish round 1 will be eligible to finish round 2, and those who finish round 2 will be eligible to finish round 3. The participants will have up to 2 weeks to finish each round, and they will receive a reminder email once after one week, as well as another reminder email 24 h before the round is over. The data will be analyzed making use of descriptive statistics, presented making use of measures of central tendency, and displayed as graphs whenever it is required to do so. In order to uncover any potential patterns in non-response, missing data will be investigated across all outcome domains (e.g., by panel, or other characteristics). If we see a pattern at the item level (for example, more than 10% of responses are absent), we will compare this information to the qualitative feedback that participants have given us.

Round 1: Using a numeric rating scale with nine points, participants will be asked to assign a rating of absolute importance for each outcome domain for “inclusion in future laparoscopic hiatal hernia repair studies” with a scale. The range of the scale is as follows: 1–3 for “of limited importance,” 4–6 for “important,” and 7–9 for “critical” [27]. Participants will be asked to rate the relative importance of each outcome domain. In addition to this option, there will also be one that says “unable to rate.” Participants are given the opportunity to elaborate on their decisions and provide extra qualitative comments and feedback that will be taken into consideration in later rounds.

A significant reduction in the number of outcome domains is one of the primary goals of the Delphi study, which seeks to reach a consensus on a core domain set consisting of a minimum number of outcome domains for the COS. Therefore, a bespoke grading system will be adopted to provide greater clarity where participants from different sub-groups either agree or disagree in their judgments [28, 29]. This system will be used in the development of a COS for laparoscopic hiatal hernia repair in patients with hiatal hernia (COS-LHHR). This technique establishes clear criteria and decision procedures for dealing with varying levels of consensus per outcome domain, such as little or no consensus (grade C or D), uncertainty (grade A and B), and unambiguous consensus (grade A** and A*). In the second round, we will only consider those outcome domains that received the highest ratings from either of the two expert panels (Table 3).

**Table 3:** Grading system for determining consensus in round 1 of the e-Delphi study

Grade	Criteria for judging agreement	Decision rule
A**	The median rating for both expert panels is 9	Consider in round two
A*	≥ 70% of respondents in each panel evaluate a certain outcome domain ≥ 7	Consider in round two
A	The median rating for a particular outcome domain is ≥ 7 for both expert panels	Include in round 2 if one of the panels earns a median score of 9 OR qualitative evidence justifies continued consideration
B	Only one expert panel had a median rating for an outcome domain ≥ 7	Include in round 2 if the median score for this group is 9 OR qualitative evidence justifies further consideration
C	The median rating for the two panels together falls between 4 and 6, and no single panel has a median rating of ≥ 7	Omit from round two (unless strong qualitative evidence supports further consideration)
D	The median rating for the two panels combined falls between 1 and 3, and no single group has a median rating of ≥ 7	Omit from round two (unless strong qualitative evidence supports further consideration)

“*” indicates different levels of A grade, with more * indicating a higher level of A grade

Rounds 2–3: A summary of responses to the previous round will be provided (individual and group median scores for each outcome). The experts will be asked to reflect on the feedback and rate again the importance of each outcome in research to indicate the rationale for their decisions and any changes. The experts will use the 9-point numerical rating scale used in the first round to score the 2 and 3 rounds.

Over three rounds, we expect to identify categories of (1) most important “core” outcomes agreed by most panelists (> 70% rated 7–9), (2) less important outcomes agreed by most panelists (> 70% rated 1–3), and (3) those where there is partial or no agreement across panelists.

### Stage 4: Consensus meeting

The purpose of this meeting is to ratify a final core set of outcomes and to recommend the COS for the laparoscopic hiatal hernia repair in patients with hiatal hernia (COS-LHHR).

This consensus gathering will include health professionals and patients who participated in the e-Delphi trial. Before the meeting, participants will get a packet of information defining the meeting’s objectives, which will include a list of the outcomes to be examined for the COS-LHHR.

Consensus for inclusion will be as follows: if ≥ 70% of panelists vote in favor and fewer than 15% of panelists vote against. Those outcomes that are deemed feasible by ≥ 70% of panelists will meet consensus for inclusion into the COS.

The meeting will consist of three sections:

- First of all, we will inform all stakeholders of the scoring criteria of the Delphi method and give a detailed explanation and introduction to the set of outcomes formed through the Delphi method. After the initial presentations, participants will discuss each outcome domain taking into account evidence of quality, acceptability, practicality, and significance.
- At the conclusion of discussion, participants will complete an anonymous survey to confirm the inclusion of each outcome category (Yes/No/Don’t Know). Agreement will be defined as 70% of participants endorsing a particular result domain.
- If there is consensus, no further discussion will be necessary. Subsequent conversations will center on disagreements and areas requiring additional elaboration.

Throughout the meetings, written notes will be taken, and votes will be recorded.

This consensus meeting will result in the ratification of a COS for laparoscopic hiatal hernia repair in patients with hiatal hernia (COS-LHHR), identifying both the core outcome domains that should be minimally assessed as well as evidence-based recommendations on the best available evaluation methods.

#### Dissemination

In accordance with the recommendations, continuous work will be required to maximize the COS’s adoption and implementation [30]. Through close coordination with our hiatal hernia group and interaction with our advisory council, we will endeavor to aggressively address this challenge.

Following the completion of each phase, a summary document will be distributed to participants to inform them of the project’s results. This will be emailed directly to the participants. COS users (including funders and journal editors) will be contacted through a variety of means, including extensive publishing and national conference presentation distribution.

## Discussion

Recent studies have highlighted the need for a standardized outcome reporting for hiatal hernia, but no such consensus is currently available yet [31–33]. The development and adoption of a core outcome set for laparoscopic hiatal hernia repair (COS-LHHR) will ensure that outcomes deemed important by key stakeholders are included in clinical trials, thereby contributing to the development of an evidence base that can be synthesized and examined more thoroughly to support healthcare decisions.

A well-developed COS-LHHR will address the current challenges associated with heterogeneity in outcome selection and reporting in research of laparoscopic hiatal hernia repair by enhancing opportunities for evidence synthesis [6, 7, 34], enabling comparative reviews of care provision across laparoscopic hiatal hernia repair, reducing reporting bias. To guarantee that these benefits are realized, continued work will be required to ensure that COS-LHHR is adopted and implemented. This will involve maximizing the COS’s diffusion and showcasing its utility in clinical practice and research.

## Data Availability

Not applicable.
